# Education and employment status among adults with Loeys-Dietz syndrome and vascular Ehlers-Danlos syndrome in Norway, a questionnaire based study

**DOI:** 10.1371/journal.pone.0279848

**Published:** 2022-12-30

**Authors:** Heidi Johansen, Gry Velvin, Ingeborg B. Lidal

**Affiliations:** 1 TRS National Resource Centre for Rare Disorders, Sunnaas Rehabilitation Hospital, Nesodden, Norway; 2 Faculty of Health, Oslo Metropolitan University (OsloMet), Oslo, Norway; University of Jyvaskyla, FINLAND

## Abstract

**Objectives:**

To describe education level and employment status among adults with Loeys-Dietz syndrome and vascular Ehlers-Danlos syndrome, and explore factors related to work participation.

**Materials and methods:**

Cross-sectional postal survey in 2018. Individuals with molecularly verified diagnosis were recruited through a National Resource Centre for Rare Disorders. A study specific questionnaire included topics on disease burden and validated instruments regarding education level, employment, pain, fatigue, psychological distress, and satisfaction with life.

**Results:**

Fifty persons (56% women) aged 18–67 years, participated. Almost 60% reported education level ≤13 years. Two thirds (66%) received disability benefits, 21 (42%) had full-time disability pension. The median age at ending work was 41 years. Full-time employed and students were younger (p = 0.014), less fatigued (p = 0.035), had less sleep problems (p = 0.028) and higher satisfaction with life (p<0.001) than those who received disability pension. A third (32%) were currently or used to be in sedentary work, and 68% currently had or used to be in practical work requiring much standing and walking (23%), much walking and lifting (34%) or heavy manual work (11%).

**Conclusions:**

There is a potential that more adults with these diagnoses can sustain employment for more years. Health and social service follow-up routines and future studies should include details on employment perspectives to reveal those at risk of poor employment and to identify modifiable factors for work participation.

## Introduction

Loeys-Dietz syndrome (LDS) and vascular Ehlers-Danlos syndrome (vEDS) are potentially life-threatening diseases within the umbrella term of rare hereditary thoracic aortic diseases (HTADs). The most serious medical complications are vascular events. LDS hallmarks are early and aggressive aneurysms and dissections of the aorta and/or other large arteries [1]. vEDS hallmarks include arterial, intestinal and/or uterine fragility with risk of rupture of internal organs in addition to risk of aneurysm and dissection of middle-sized arteries [2]. Other organs affected commonly include the musculoskeletal system, craniofacial structures, and cutaneous features [3]. Both diagnoses are suspected on basis of family history or a clinical history of arterial rupture, dissection or aneurysm, rupture of the large intestine, or specific pregnancy complications [3]. Since there are clinical overlap between HTADs, the diagnoses should be confirmed by identification of pathogenic gene variants to allow for appropriate surveillance, treatment, and family studies [3]. LDS is caused by a mutation of genes encoding for transforming growth factor-beta signalling pathway *TGFBR1*, *TGFBR2*, *SMAD3*, and *TGFB2* [1]. vEDS results from pathogenic variants in *COL3A1*, encoding type III collagen that is the major expressed collagen in blood vessels and hollow organs [2]. The prevalence of these diseases worldwide is highly uncertain; for LDS the prevalence is unknown [1] and for vEDS the prevalence is estimated 1: 50–200.000 [2].

Most individuals with LDS or vEDS have family members with the same HTAD condition, and many have experienced close relatives being critically ill or even die at an early age [4]. A wide spectrum of clinical variability and heterogeneity of cardiovascular involvement is seen among individuals with these gene defects [3]. Typically, persons with HTADs are recommended annual specialist cardiovascular evaluation, or follow-up intervals as clinically indicated. To reduce the risk of major vascular events, anti-hypertensive medication is prescribed to assure that blood pressure is maintained in the normal range, and sometimes prophylactic aortic surgery is scheduled [1, 2, 5]. Furthermore, it is advisable to modulate lifestyle to lower the risk of cardiovascular stress, ruptures and to reduce musculoskeletal complications. A clear understanding of possible implications of HTADs is important to everyday living and employment. A person’s career decision-making and career options may be influenced by cardiovascular recommendations, specifically advises to avoid stress and heavy work to reduce hypertensive episodes, but also by prophylactic surgery or vascular events. Furthermore, musculoskeletal issues and pain may influence on job demands. Thus, the disease burden may affect the risk of early retirement [4].

As far as we know, no previous research has investigated education and employment among people with LDS or vEDS. However, several studies found reduced employment rate among adults with Marfan syndrome [6–8], and in a mixed HTAD population [9]. Findings related to employment status: Velvin et al. found a significant association between severe fatigue and disability pension [7]. Goldfinger et al. reported significant association between increased physical health related quality of life and employment [8], Rao et al. pointed to high absenteeism due to reduced health and hospitalizations due to typical health problems related to Marfan syndrome [6]. Thijssen et al. reported significant associations between increased quality of life and employment among HTAD participants [9].

The School system in Norway is expected to give students vocational guidance to do suitable career choices and prepare for paid work. NAV (the Norwegian Labour and Welfare Administration) also has several schemes aimed at those who are at risk of dropping out of working life, including retraining programs and financial support (benefits) [10]. In Norway, the overall employment rate among persons aged 15–74 years is high (approximately 58% full-time employed or students), many work part-time (17% with or without disability benefits). The unemployment rate is low (4%), and approximately 10% receive full-time disability pension [11]. Factors affecting work participation in the general population are complex: Fewer women than men are employed [11], low levels of education [11], reduced mental- and physical health [12], chronic musculoskeletal pain [13] and fatigue symptoms [14] are found to be associated with low work participation.

In a previous paper, we showed that a considerable group of LDS and vEDS participants left work before retirement age [4], and many scored to be dissatisfied with their vocational situation [15]. With data collected from the same study group, the aim of the current paper was to present a more detailed description of educational level and issues related to work participation (degree of physical demands in work, and work challenges). Another aim was to explore the association between work participation and demographic and clinical factors in this study group of adults with LDS and vEDS.

## Materials and methods

### Design

**Fig 1:**
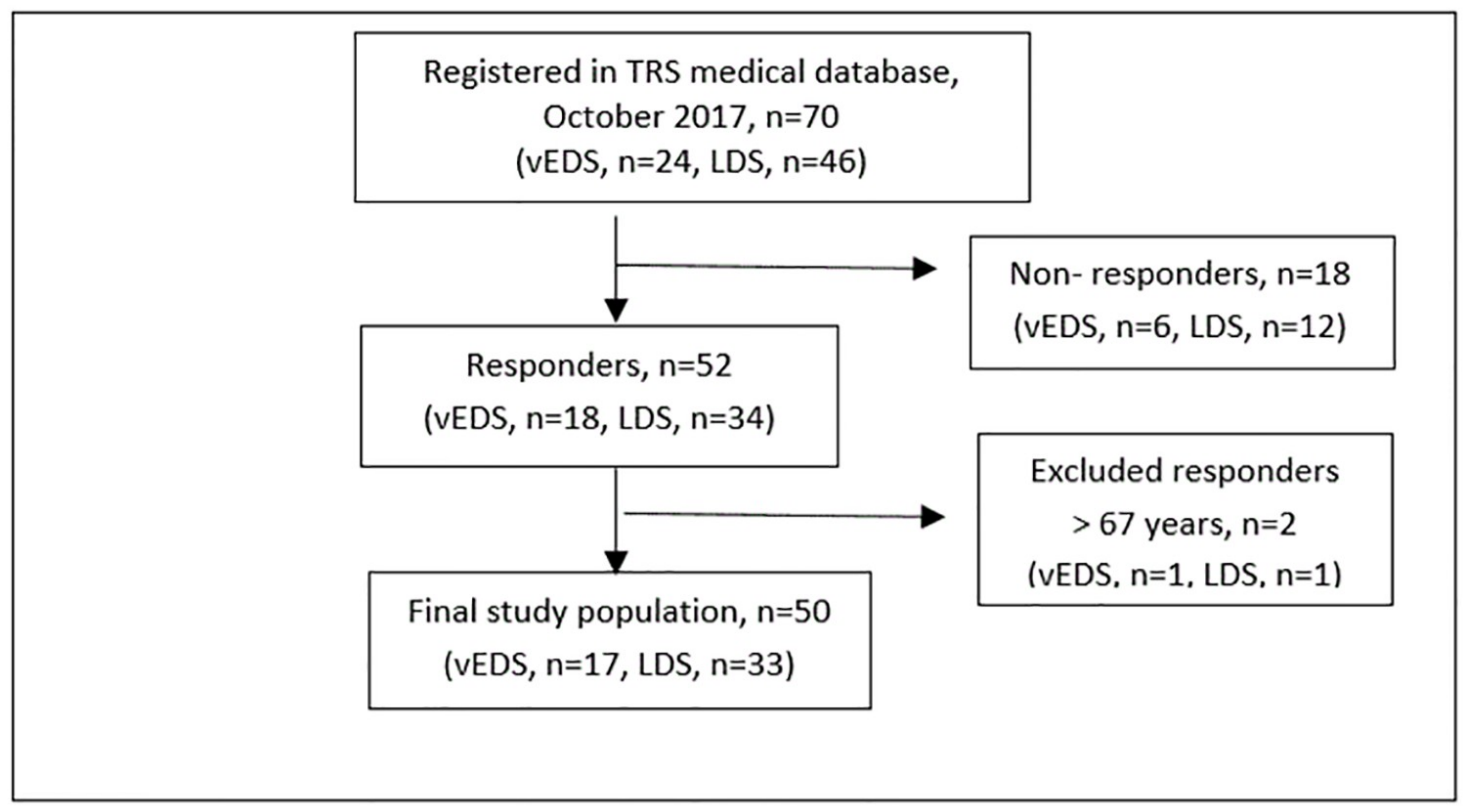
Flowchart of inclusion. Abbreviations: LDS = Loeys Dietz syndrome, vEDS = Vascular Ehlers-Danlos`syndrome, TRS is a National resource center for rare disorders. Total response rate was 74%, 75% for the vEDS group and 74% for the LDS group.

This survey was part of a larger study on physical function and psychosocial aspects in adults with HTADs [4, 15–19]. In this paper, we used data from a cross-sectional quantitative, questionnaire-based study of individuals with LDS and vEDS of working age (18–67 years) (n = 50). The inclusion process is presented in a flow chart (Fig 1).

### Participants and recruitment

In January 2018, all patients (n = 70), aged 18 years and older, with molecularly verified LDS or vEDS, registered at the TRS National Resource Centre for Rare Disorders [20] in Norway were invited to participate. Eligible individuals received a letter with study information, a consent form to be signed, the questionnaire and a prepaid return-addressed envelope. The non-responders received a written reminder three weeks later.

### Questionnaire

A study-specific questionnaire was developed in cooperation with representatives from the Norwegian Marfan- and Loeys-Dietz Association and the Norwegian Ehlers-Danlos Association. Participants were asked to provide information on:

**Demographic factors**: Gender, age, region of residence, parenthood (yes/no), family member(s) with LDS or vEDS (yes/no), age at diagnose confirmation, years of education (≤13 years *or* >13 years). Work participation was investigated with questions gathered from “The Labor Force Survey” in Norway [11], and included: 1) present employment status, i.e. *full-time employed or students*; *disability benefits* = full-time or part-time disability pension (> 50%) or retraining programs, (yes/no), 2) age at discontinued working if relevant, 3) level of physical demanding work by four categories: a) sedentary work, b) work that requires much walking and standing, c) work that requires much walking and lifting, d) heavy manual work), and 4) Study specific questions on educational or work challenges and related adjustments (yes/no). These included questions about whether the LDS/vEDS diagnosis had impacted education and/or choice of work; needed vocational guidance; paid work during education; received additional scholarship; whether the workplace was informed about the LDS/vEDS diagnosis; experience of being met with understanding at work; pain problems as an obstacle to work ability; LDS/vEDS diagnosis influenced reason for retirement; could have continued to work, if the work had been adjusted to suit my health situation better.

**Clinical factors**: A history of aneurysm/dissection of the aorta *and/or* of other arteries; undergone acute/prophylactic vascular surgery *and/or* mitral valve surgery; a history of impaired vision and/or hearing, hernia, neck instability, organ rupture, pneumothorax, scoliosis, affected skin, joint, and asthma/allergy (yes/no). To explore health burden, two sum-scores were calculated on the basis of the above mentioned number of self-reported health problems: *Cardiovascular burden* (0–7) and *Multi organ symptom burden* (0–11) [4]. For clinical interpretation, persons were classified with high levels of cardiovascular- and/or multi organ symptom burden if they reached the study group’s median score or above, i.e. 3.0 and/or 4.5, respectively.

*Pain* was measured with one item from the Standardized Nordic Questionnaire (SNQ) [21]: Have you experienced chronic musculoskeletal pain lasting more than three months, during the last year? (yes/no).

*Fatigue* symptoms were measured with the Fatigue Severity Scale (FSS) [22], a nine-item questionnaire, rated on a 7- point scale; higher score indicates higher levels of fatigue. Cut-off values for moderate to severe fatigue symptoms = FSS mean score >5, low fatigue symptoms = FSS mean score ≤ 5 [23]. FSS has been found valid and reliable [22, 23].

To study *psychological distress*, the Hospital Anxiety & Depression Scale (HADS) was used [24]. HADS consists of two subscales, HADS-A measuring anxiety, and HADS-D measuring depression, with 7 items each rated on a 4-point scale (0–3). A score of < 8 points within each of the subscales indicates normal range. Higher scores indicate more anxiety/depression symptoms; 8–10 mild, 11–14 moderate, 15–21 severe symptoms of anxiety/depression [24]. We used a score of 8 as cut-off, since this is commonly used as an indication to initiate further clinical investigation [24]. The Norwegian version of HADS is well-validated [25].

*The SWLS* (Satisfaction With Life Scale) provides a well-validated global measure of satisfaction with life as an overall summation [26]. It consists of five questions rated on a 7-point Likert scale, from ‘strongly agree’ (option 7) to ‘strongly disagree’ (option 1). In this study the scores were summed to a total score ranging from 5 to 35. A score of 20 represents the midpoint between satisfied and dissatisfied with life [27].

*Sleep problems* were measured with one study specific overall question and five sub-questions: Do you have sleep problems (yes/no), if yes: do you have: a) problems with falling asleep, b) sleeping coherently, c) with early wake up, d) stay awake during the day and e) sleep medication use the last four weeks (yes/no).

### Data analysis

Descriptive statistics are presented as frequencies, proportions, range and means with standard deviation (SD). Due to a large clinical overlap between the two diagnostic groups (LDS and vEDS), we have chosen to pool the groups in the analyses to gain better statistical power in this study.

To explore differences between the two subgroups *full-time employed or students* and *disability benefits* (full-time or part-time disability pension (>50%) or on retraining programs) Fisher`s exact test was used to compare categorical variables and Independent Samples *t*-test was used to compare continuous variables. Multivariate analyses were considered inappropriate due to the small sample size. Because there were few missing data, no imputation method was used. The exact n for each of the analyses are given in the tables. The statistical analyses were conducted by using SPSS Statistics for Windows version 26. A statistical significant level was set at *p* ≤ 0.05.

### Ethics

The Regional Ethics Committee for Medical and Health Research Ethics in southeastern Norway (No. 2017/745) approved the study. Written information on the aim of the study, voluntariness to participate, the opportunity to withdraw from the study and written informed consent was obtained from all participants. The results are reported in accordance with the STROBE guidelines for observational studies [28] (S1 Table).

## Results

### Demographic and clinical characteristics

**Fig 2:**
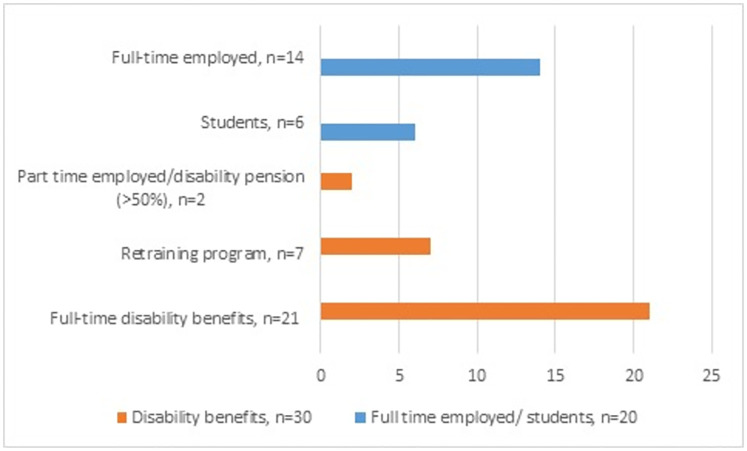
Distribution (%) of employment status, fulltime employment/students vs. disability benefits.

**Fig 3:**
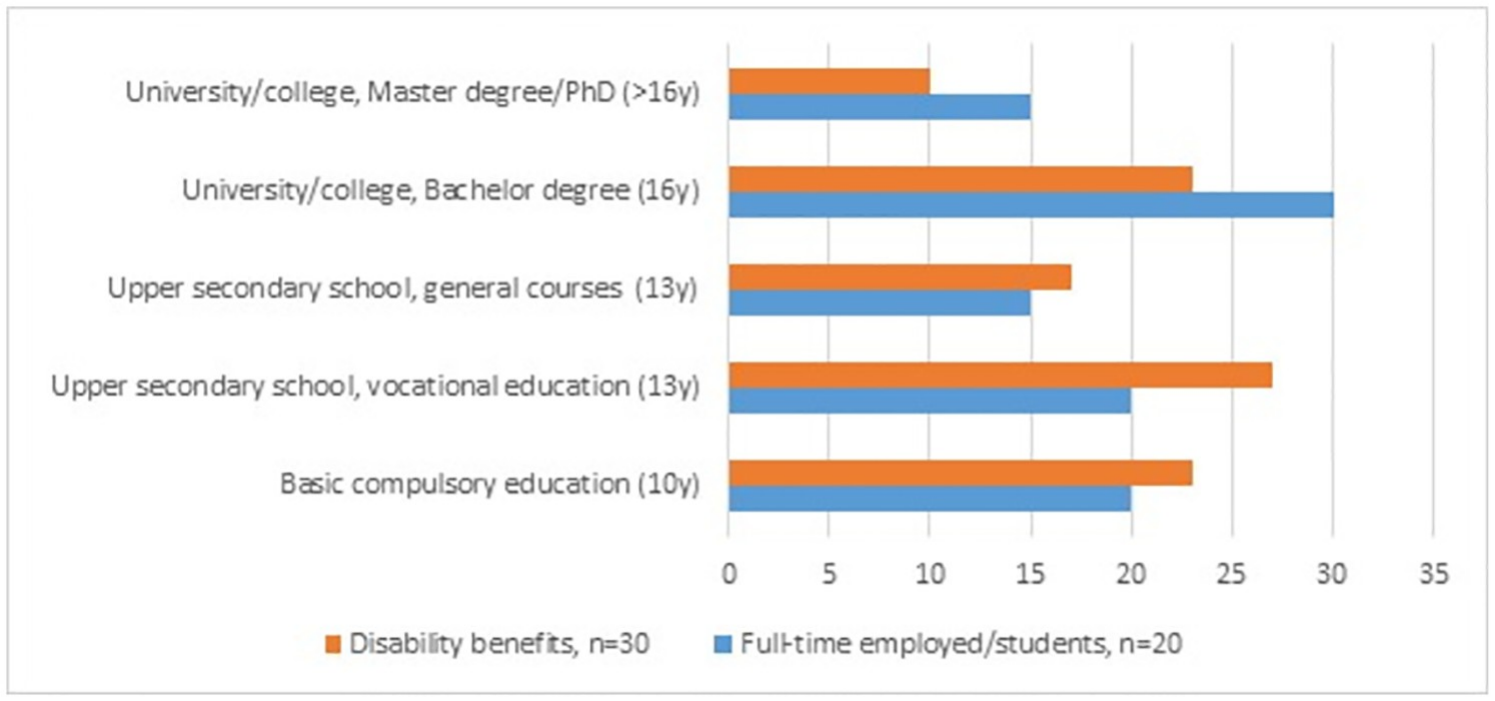
Distribution (%) of educational level according to employment status.

Fifty persons with LDS (n = 33) and vEDS (n = 17) of working age answered the questionnaire. Table 1 shows their demographic and clinical characteristics according to employment status, *full-time employed or students* (n = 20) and *disability benefits* (n = 30) (full-time disability pension, part-time disability pension (>50%) and work, retraining programs). The median age was 41.5 years, a slight majority were women, and more than half of the participants lived together with a partner and had children. The median time since the confirmation of the diagnosis was 6.5 years. Disease burden was high; cardiovascular burden (60%), multi-organ burden (34%), chronic musculoskeletal pain (80%), fatigue symptoms according to the FSS scores >5 (58%), and many reported symptoms of psychological distress (94% had HADS-A score ≥8, 76% had HADS-D score ≥8). Those who were *full-time employed or students* were younger (p = 0.014), fewer reported fatigue symptoms above the FSS threshold of 5 (p = 0.035), sleep problems were less common (p = 0.028), and more reported better satisfaction with life (p>0.001) compared to those who received *disability benefits*. Fig 2 shows a more detailed distribution of employment status, and Fig 3 illustrates the education level in those full-time employed or students and in those who received disability benefits. The median age of early retirement (i.e. receiving full-time disability pension), was 41 years.

**Table 1:** Demographic and clinical factors in 50 adults with Loeys-Dietz- syndrome or vascular Ehlers-Danlos syndrome, according to employment status.

Characteristics	Total study population, n = 50	^a^Full-time employed/students n = 20	^b^Disability benefits n = 30	Difference p-value
Diagnosis, LDS, n(%)	33(66)	13(65)	20(66)	0.570
Gender, women, n(%)	28 (56)	10(50)	18(60)	0.342
Median age (range) year	41.5 (18–67)	33.0(18–66)	47.0(23–67)	0.014
Living with partner, n(%)	27 (54)	8(40)	19(63)	0.091
Parenthood, n(%)	25(50)	7(35)	18(60)	0.074
Family member with corresponding diagnose, n(%)	43 (86)	17(85)	26(86)	0.590
Median age (range) discontinued working (received full disability pension) (n = 19)			41.0(23–65)	
Median age (range) for diagnose confirmation	33.5(6–33)	29.5(6–57)	36.5(7–63)	0.217
Formal education level >13 years, n(%)	19 (38)	9(45)	10(33)	0.295
Chronic musculoskeletal pain, n(%)	40 (80)	15(75)	25(83)	0.355
Mean fatigue score≥5 (FSS), n(%)	29(58)	8(40)	21(70)	**0.035**
Sleep problems, n(%)	34(68)	10(50)	24(80)	**0.028**
^d^High cardiovascular burden level (median score ≥ 3), n (%)	30(60)	9(45)	21(70)	0.100
^e^High multi organ burden level (median score ≥4.5) n (%)	17(34)	6(30)	11(37)	0.446
^f^ HADS-A score ≥8, n (%)	47(94)	20(100)	27(90)	0.207
^f^ HADS-D score≥8, n (%)	38(76)	18(90)	20(66)	0.080
^g^Satisfaction with life scale (SWLS) median (range) score	23.0 (6–35)	27.0 (17–33)	18.0 (6–35)	**<0.001**

Abbreviasjons: vEDS = Vascular Ehlers-Danlos syndrome; LDS = Loeys-Dietz syndrome

^a^Employed included persons who were full-time employed (n = 14) or students (n = 6) (without any disability pension). Disability benefits: disability pension (n = 21), retraining programs (n = 7) or part-time disability pension/employed (n = 2)

^d^Number of cardiovascular burdens (0–7): aorta-aneurism, aorta-dissection, other aneurism, other dissection, aorta-surgery (acute, prophylactic or both), mitral valve surgery, use of antihypertensive medication

^e^Number of multi-organ burdens (0–11): impaired vision and/or hearing, neck instability, pneumothorax, hernia, organ rupture, scoliosis, skin problems, joint problems, allergies, and stomach discomfort.

^f^Hospital Anxiety & Depression scale: anxiety (HADS-A) and depression (HADS-D) subscale

^g^ Satisfaction with life scale (SWLS), score range 5–35, higher score indicate higher satisfaction with life

A statistical significant level was set at p ≤ 0.05.

### Work requirements and work adjustments

**Fig 4:**
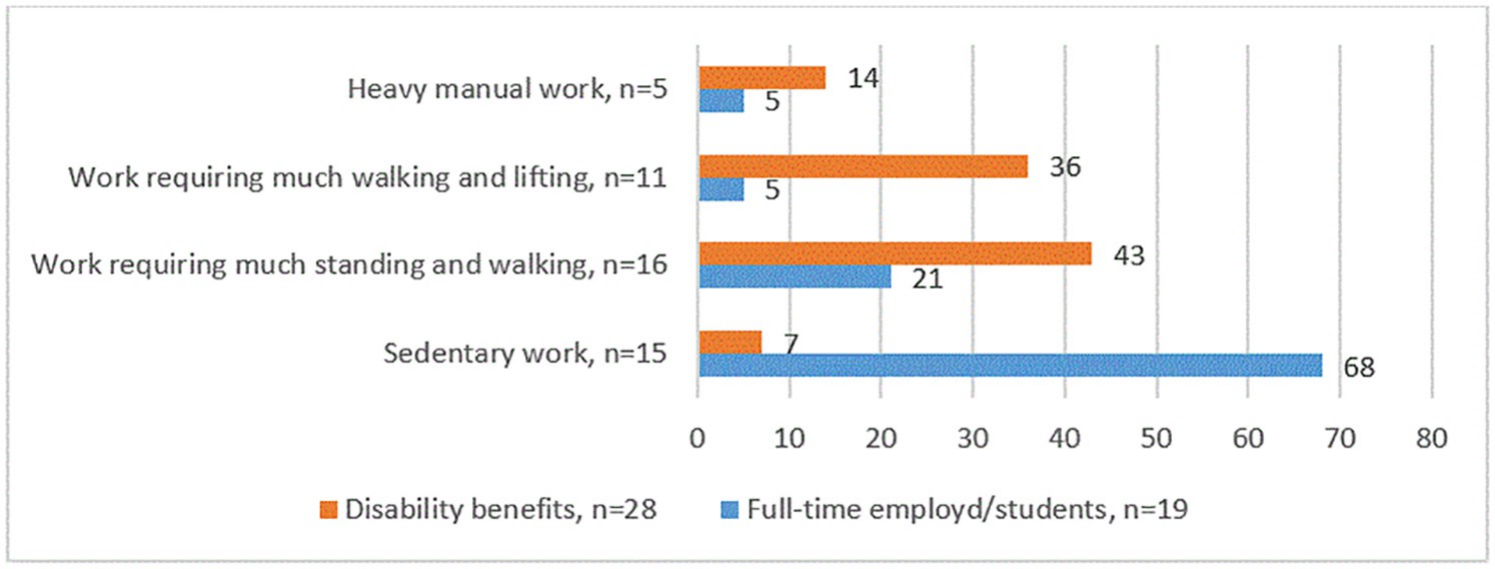
Proportion (%) of those currently working full-time versus those receiving disability benefits according to type of work (sedentary vs. different degrees of physical demanding). Three reports missing (one persons never been employed, two missing).

Fig 4 shows the level of experienced physical demands in work situation according to employment status: full-time employed/students compared to those receiving disability benefits who had been in previous work. About one third (32%) of all the participants currently had or used to be in sedentary work, and 68% currently had or used to be in practical work requiring much standing and walking (23%), much walking and lifting (34%) or heavy manual work (11%). Most (68%) of those who were full-time employed or students had sedentary work. Nearly all (93%) of those who received disability benefits had earlier physically demanding work; 12 persons with work that required standing and walking, ten used to have work requiring walking and lifting, and four used to have heavy manual work.

**Fig 5:**
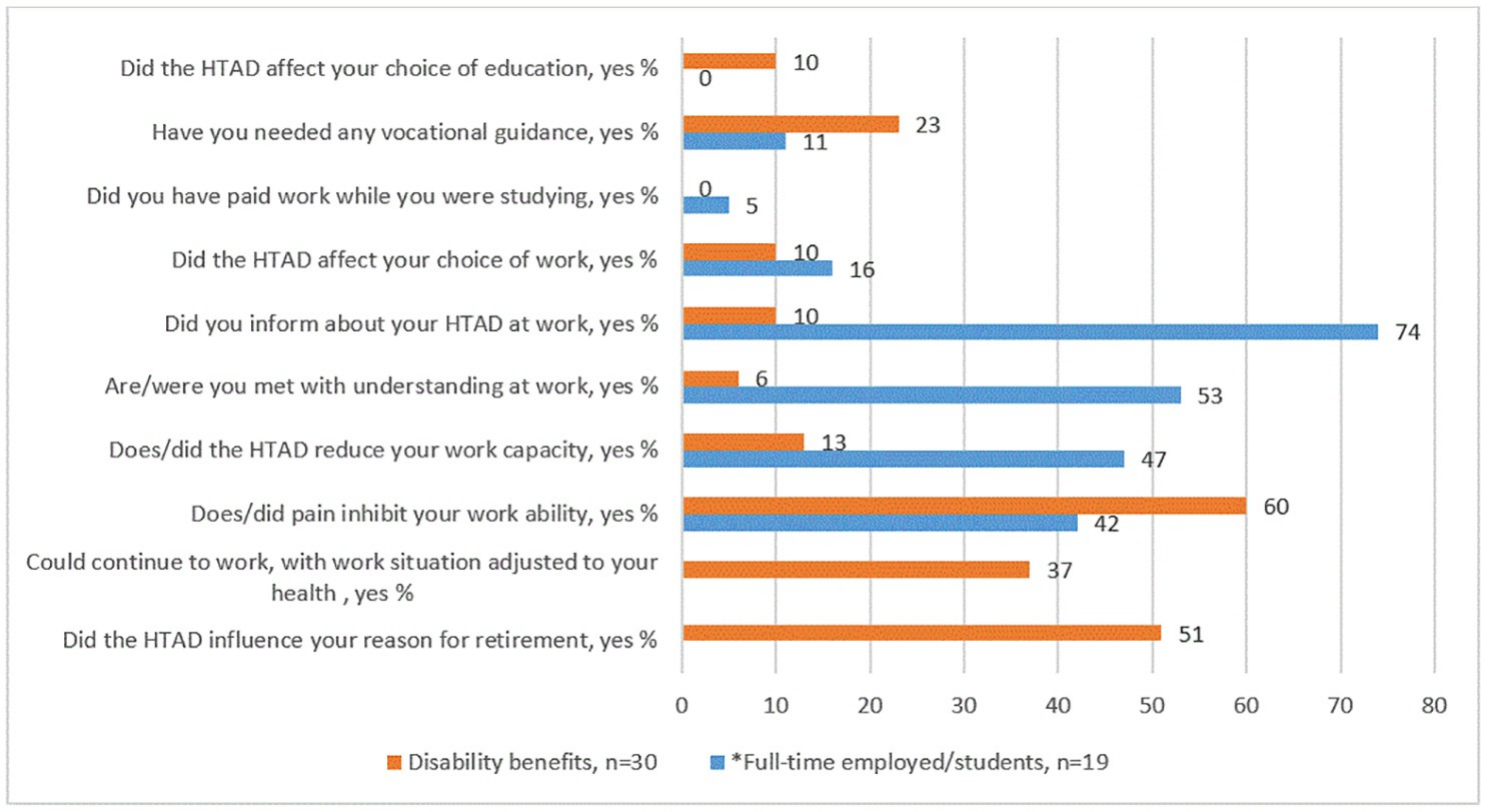
Self-reported work challenges and work adjustments according to employment status. *One missing.

Fig 5 shows self-reported work challenges and work adjustments according to employment status. Those who received disability benefits, recalled and reported their experiences from the period they were employed. Compared to persons who received disability benefits, a higher proportion of those currently full-time employed/students had informed about their HTAD diagnose at work (10% vs. 74%). They had also been met with understanding at work (53% vs. 6% of those who received disability benefits). Forty-two percent reported pain problems as an obstacle to work (vs. 60% of those who received disability benefits), and few (11%) had needed vocational guidance (vs. 23% of those who received disability benefits). Half of the participants who received disability benefits reported that the decision to retire had been influenced by their health situation with the HTAD diagnosis and 37% claimed that they might have been able to continue to work if necessary adjustments at work had taken place.

## Discussion

### Main findings

In this study of Norwegian adults with vEDS or LDS of working age, two thirds were early retired (i.e received disability benefits). The median age at ending work (receiving full-time disability pension) was 41 years. In the total study group, approximately one third used to be in sedentary work, 57% in practical work (much walking, lifting) and 11% in heavy manual work. Almost 40% of those who received disability benefits claimed that they could have sustained in work with necessary workplace adjustments. Our results indicated that those who were full-time employed or studying had less fatigue-symptoms, fewer had sleep problems, and they reported to be more satisfied with their lives compared to participants who received disability benefits.

### Education, career choice and work participation

According to our experiences, facilitating career and counselling career development or transition in persons with HTADs are necessary initiatives that may help these patients to successful and sustainable employment. During the diagnostic process, people with HTADs usually receive lifestyle advices, such as restrictions on physical activity and heavy physical work, to reduce the risk of high blood pressure and excessive stress on fragile tissues [16, 17, 29]. Furthermore, persons with HTADs often require prolonged absences from work to convalesce after acute disease events [6]. Therefore, professional guidance on employment barriers and support to return to work should be a natural part of the follow-up of persons with HTADs as well as in persons awaiting to be diagnosed.

Although LDS and vEDS are genetic disorders, many study participants were not diagnosed until years after graduation or after several years in working life. In this study group, the median age at diagnosis was 33.5 years. Thus, many had made career decisions without assessing potential HTAD issues, and few reported to have needed vocational guidance. Some of our participants (n = 7) stated that they were undergoing retraining. We assume that more people could have benefited from retraining for occupations that are less physically demanding and/or mentally stressful.

### Workplace adjustments and health issues

Employment outcomes are results of complex interactions between disease-related and contextual (personal/environmental) factors. Modifiable factors are those that may be amenable to interventions (through improvement or preventing deterioration). When it comes to disease burden and musculoskeletal pain, the study findings did not indicate statistically significant differences between full-time employed or students and those who received disability benefits. In total, 80% reported chronic pain and many participants in both groups reported that pain influenced their work ability. However, fatigue and sleep problems seemed to be more common among those who received disability benefits. This corresponds with findings by Velvin et al. who studied adults with Marfan syndrome and found a significant association between low work participation and severe fatigue [7]. Furthermore, about 40% of those who received disability benefits claimed that they could have continued to work if the work situation had been better adjusted to their health situation. Commonly, those who worked full-time or were students had informed their manager and colleagues about their disease and about activity restrictions they had been recommended. Surprisingly, more than 20% of them reported to not have been met with understanding of these problems. We do not know how the disease information was communicated and by whom, but certainly the role of the employer is a modifiable factor (among many) to achieve sustainable employment.

In a previous article on life satisfaction in this study population [15], we revealed that a large proportion (74%) of the participants reported to be dissatisfied with the working life domain. Another significant finding was the association between more fatigue symptoms and less satisfaction with work life. Fatigue symptoms seems to be a challenge for people with HTADs [19, 30], also in relation to work life. From our experiences, we see that it is difficult to find good adaptations that can alleviate fatigue symptoms in working life. In HTAD conditions, several studies have found a connection between better quality of life reports and work participation [6, 8, 9]. This is in line with the current finding of higher SWLS scores among full-time employed or students compared to those who received disability benefits. We agree with the explanation of Thijssen et al. who claimed that work participation affects social life, family life, self-esteem, independence and the economic situation [9], thus it is understandable that work participation can affect the experience of life satisfaction in HTAD patients. Additionally, Rao et al. pointed to considerable absence from work among adults with Marfan syndrome due to severe health problems and necessary hospital stays [6]. Persons with LDS and vEDS also have multiple health problems overlapping with those of patients with Marfan syndrome [4, 7, 30]. The need of medical follow-up, major surgery, need for convalescence, and rehabilitation may entail significant absence in both education and work among persons with these diagnoses. Thus, future studies should explore the complex interactions between disease-related and contextual factors in HTADs to determine which persons are at risk of poor employment outcomes. Especially, it would be important to identify modifiable factors that may reduce the time away from work after major surgery. Furthermore, employment is typically a secondary outcome of studies, and we want to encourage these to include more details on this important theme.

### Strength and limitations

The main strengths of this study were the high response rate, the inclusion of persons with a genetically verified LDS or vEDS, and the completeness of the data with very few missing reports. Importantly, participants were residents from all regions of Norway and registered at a National Resource Centre for Rare Disorders, which is a free of charge service for all citizens with HTADs. However, registration at the center is voluntary, and therefore it is not a registry covering the total population with HTADs in Norway. Furthermore, individuals are usually registered because of their need for services such as diagnosis information, advices on lifestyle changes, or counselling on welfare services. Persons choosing to register may therefore differ from those who do not. Additionally, due to the rarity of the diagnoses, no available national registry and unsure prevalence worldwide, we do not know the representativeness of the 52 participants from the total population of LDS or vEDS in Norway (general population of 5.3 million inhabitants).

The study questionnaire included instruments that are well proven and validated in several patient populations, however as far as we know, not among HTADs. The self-reports might have introduced recall bias to questions on retrospective information.

The relatively small sample size leads to low statistical power for several analyses. Our findings do not allow for firm conclusions or generalizability to LDS or vEDS patients as such, and there are a need for more robust studies.

## Conclusions

In this study of Norwegian adults with LDS or vEDS in working age, two thirds were early retired (received disability benefits) and many reported that they could have sustained employment with better adaptations. Very few had taken their HTAD into account when making decisions about education or with type of work. A delayed diagnosis confirmation may be one reason, as age at diagnosis ranged from six to 33 years. Approximately two thirds used to be occupied in non-sedentary work. When it comes to disease burden and musculoskeletal pain, no statistically significant differences were found between full-time employed persons and those not employed or part-time employed. Although 80% reported chronic pain and many participants reported that pain influenced their work ability, fatigue and sleep problems seemed to have greater impact on work participation. Higher satisfaction with life scores were found among those full-time employed or studying. We suggest that health-service follow-up routines and future studies of HTAD groups should include details on employment perspectives to reveal those at risk of poor employment and to identify modifiable factors for work participation.

## Supporting information

S1 Table STROBE statement—Checklist of items that should be included in reports of observational studies. (DOCX)
